# Prognostic Significance and Immune Landscape of a Cuproptosis-Related LncRNA Signature in Ovarian Cancer

**DOI:** 10.3390/biomedicines12112640

**Published:** 2024-11-19

**Authors:** Min Zhou, Jianming Tang, Guotao Huang, Li Hong

**Affiliations:** Department of Gynecology and Obstetrics, Renmin Hospital of Wuhan University, Wuhan 430060, China; zhoumin2022@whu.edu.cn (M.Z.);

**Keywords:** ovarian cancer, cuproptosis, lncRNA, tumor microenvironment, prognosis, immunotherapy

## Abstract

**Background:** Cuproptosis is a copper-induced mitochondrial cell death, and regulating cuproptosis is becoming a rising cancer treatment modality. Here, we attempted to establish a cuproptosis-associated lncRNAs (CRLs) signature (CRlncSig) to predict the survival, immune landscape, and treatment response in ovarian cancer (OC) patients. **Methods:** A series of statistical analyses were used to identify the key CRLs that are closely related to the prognosis, and a prognostic CRlncSig was constructed. The predictive accuracy of the CRlncSig was further validated in an independent Gene Expression Omnibus (GEO) set. Then, we compared the immune cell infiltration, immune checkpoints, tumor microenvironment (TME), tumor mutational burden (TMB), drug sensitivity, and efficacy of immunotherapy between the two subgroups. We further built a nomogram integrating the CRlncSig and different clinical traits to enhance the clinical application of the CRlncSig. **Results:** Nine hub CRLs, namely RGMB-AS1, TYMSOS, DANCR, LINC00702, LINC00240, LINC00996, DNM1P35, LINC00892, and TMEM254-AS1, were correlated with the overall survival (OS) of OC and a prognostic CRlncSig was established. The CRlncSig classified OC patients into two risk groups with strikingly different survival probabilities. The time-dependent ROC (tdROC) curves demonstrated good predictive ability in both the training cohort and an independent validation cohort. Multivariate analysis confirmed the independent predictive performance of the CRlncSig. We constructed a nomogram based on the CRlncSig, which can predict the prognosis of OC patients. The high-risk score was characterized by decreased immune cell infiltration and activation of stroma, while activation of immunity was observed in the low-risk subgroup. Moreover, patients in low-risk subgroups had more Immunophenoscore (IPS) and fewer immune escapes compared to high-risk subgroups. Finally, an immunotherapeutic cohort confirmed the value of the CRlncSig in predicting immunotherapy outcomes. **Conclusions:** The developed CRlncSig may be promising for the clinical prediction of OC patient outcomes and immunotherapeutic responses.

## 1. Introduction

Ovarian cancer (OC) is a heterogeneous disease with the highest mortality rate and the worst prognosis of any gynecological malignancy [1]. Although it accounts for only 3% of all female cancers, the annual incidence of OC worldwide is estimated at 21,750 new cases and an estimated 13,940 deaths per year [2]. Given the complex molecular and genetic changes and variable responses to treatment, OC is a severe concern for women. At present, there is no screening tool for OC, and with diagnosis often occurring at later stages, metastasis and recurrence are quite high in these patients. The standard treatment for OC is surgery followed by combination chemotherapy. However, most patients in advanced stages of the disease will relapse and develop chemoresistance within a few years [3]. The 5-year survival rate for early-stage OC is 92%, which drops to around 29% for advanced-stage OC [4]. Therefore, novel markers are warranted to enhance the prognostication of OC patients and identify innovative therapeutic interventions.

Copper (Cu) is an essential transition metal that strongly influences basic cellular processes, including oxygen metabolism, mitochondrial respiration, and iron uptake [5,6]. Studies have proved that copper levels frequently increased in cancer, and that the dyshomeostasis of copper may cause cytotoxicity, altering intracellular copper levels that affect tumor progression [7,8]. Tsvetkov et al. [9] identified a unique form of regulatory cell death (RCD) that is copper-dependent and distinct from apoptosis, autophagy, ferroptosis, necroptosis, and pyroptosis. This copper-dependent death is mediated by the direct binding of copper to the lipoylated components of the tricarboxylic acid (TCA) cycle, which results in the abnormal aggregation of lipoylated protein and the loss of iron–sulfur cluster proteins, resulting in proteotoxic stress and ultimately cellular death. Cuproptosis-related genes (CRGs) could regulate tumor cell proliferation, invasion, and metastasis [10]. Given that copper drooping is more inflammatory than other types of RCD, understanding how cuproptosis is initiated and propagated may help identify cuproptosis-associated therapeutic interventions and possible combination treatments for human cancer [11]. Presently, the roles of CRGs in OC remain largely unknown and cannot be ignored.

Long noncoding RNAs (lncRNA) are RNA molecules with a transcript length of more than 200 nt that have no protein-coding potential [12]. Growing evidence has indicated the intimate relevance between the aberrant expression of lncRNAs and tumorigenesis, metastasis, and drug resistance [13,14,15]. Although many lncRNAs have been experimentally shown to be linked to cell death [16,17,18], reports evaluating the relationship between CRGs and lncRNAs in OC are lacking. Accordingly, it is an objective of this study to identify prognostic cuproptosis-related lncRNAs (CRLs) and determine the CRLs with the potential of regulating cuproptosis in OC.

In our study, we built a cuproptosis-associated lncRNAs signature (CRlncSig) to predict clinical outcomes and explored that this CRlncSig is related to immune microenvironment infiltration and immunotherapy. Our findings provide new insights into the biological functions and molecular mechanisms of cuproptosis in OC and also suggest the potential benefit of immunotherapy in OC patients.

## 2. Materials and Methods

### 2.1. Data Obtained and Analysis

The RNA sequencing data of OC tissues and normal ovarian tissues were obtained from The Cancer Genome Atlas (TCGA; https://portal.gdc.cancer.gov/, accessed on 10 August 2022) and Genotype-Tissue Expression (GTEx; https://xenabrowser.net/datapages/, accessed on 10 August 2022) databases. The data type was HTseq-FPKM and the gene expression level was processed by log2(count + 1). After integrating the data from GTEx and TCGA, 88 normal tissues and 379 tumor tissues were used for subsequent analysis. Clinicopathological traits were obtained from TCGA-OV and patients with unavailable clinical information were removed. The GSE9891 dataset (https://www.ncbi.nlm.nih.gov/geo/, accessed on 10 August 2022) was obtained from the Gene Expression Omnibus (GEO) database. The TCGA dataset was employed to build the risk model, and the GEO dataset was employed to validate the reliability of the model.

### 2.2. Screening for Cuproptosis-Related lncRNAs (CRLs)

We obtained 19 cuproptosis-related genes (CRGs) from previous literature [19] and they are displayed in Table S1. The R package “corrplot” (version 0.92) was employed to assess the correlation between the extracted lncRNAs and the CRGs derived from the TCGA-OV dataset. Furthermore, CRLs were identified based on the established criteria of *p* < 0.001 and |R| ≥ 0.3, utilizing Pearson correlation analysis for this purpose.

### 2.3. Construction and Validation of CRlncSig in OC

In the training cohort, CRLs correlated with OS were selected by univariate Cox regression analysis. Then, LASSO Cox regression analysis was conducted to select the optimal candidates. After combining the expression levels of each specific gene, we constructed a CRlncSig for each patient and weight it in a LASSO Cox regression analysis based on its estimated regression coefficient (β).

Risk score = $\sum_{i=1}^{N}Exp\times Coef.$ Exp and Coef represented the expression level and regression coefficient of corresponding CRLs, respectively. Patients were divided into low- and high-risk groups based on the median risk score. Kaplan–Meier (K–M) survival analysis was then conducted to compare the difference between survival curves. Concordance index (C-index) and area under the receiver operating characteristic (ROC) curves (AUC) were employed to evaluate the predictive accuracy of the CRlncSig. Principal Component Analysis (PCA) is a widely used tool for narrowing down and separating computer vision attributes. PCA and t-SNE analyses were performed to assess the grouping ability of the CRlncSig using the “Rtsne” (version 0.17) and “ggplot2” packages (version 3.5.1). In addition, the GSE9891 dataset was further utilized for validating the performance of the CRlncSig.

### 2.4. Building and Validation of a Prognostic Nomogram

The potential prognostic variables (age, FIGO stage, tumor size, and grade) with significant differences (*p* < 0.05) in univariate analysis were screened for the Cox regression model for multivariate analysis. Then, a prognostic nomogram was built utilizing these independent prognostic indicators. ROC analysis was performed using the “timeROC” packages (version 0.4) to verify the efficacy of the nomogram. Calibrations were plotted by comparing estimated versus observed survival.

### 2.5. TME Infiltration Level Analysis

To investigate the immune microenvironment (TME) of OC patients in different risk subgroups, the CIBERSORT method [20] was adopted to evaluate the infiltration degrees of 22 subtypes of infiltrating immune cells. We further investigated the relationship between risk score and immune cells by Spearman’s correlation analysis. Moreover, the relationship between the CRlncSig and TME score was studied by ESTIMATE algorithm [21].

### 2.6. Immunotherapeutic Response Prediction

We assessed whether common immune checkpoint activation differs between the two subgroups. The tumor immune dysfunction and exclusion (TIDE) algorithm (http://tide.dfci.harvard.edu//, accessed on 10 August 2022) was employed to predict inhibitory responses to PD-1 and CTLA4 immune checkpoints in two risk populations [22]. The possibility of patients benefiting from immunotherapy can be inferred by immunophenoscore (IPS) [23]. The IPS of TCGA-OV was obtained through TCIA (https://tcia.at/, accessed on 10 August 2022) database and employed to assess the response status of OC patients to immunotherapy. Meanwhile, an immunotherapeutic cohort (IMvigor210 cohort) was included in our study. The CRlncSig was fitted in the IMvigor210 cohort to validate its value in predicting immunotherapeutic response.

### 2.7. Correlation Analysis of CRlncSig with Tumor Mutation Burden (TMB)

The distributions of TMB among the various risk score groups were examined by “ggpubr” package (version 0.6.0). Patients were categorized into various TMB groups, and the difference in OS of patients with different mutational burdens was compared. Additionally, a combined assessment of TMB and risk scores was conducted to evaluate the survival outcomes of OC patients.

### 2.8. Drug Sensitivity Prediction

To explore the response of different risk score groups to diverse drugs, we analyzed the correlation between the semi-inhibitory concentration (IC50) of frequently utilized medicines and CRLncSig using the “pRRophetic” package (version 0.5) [24].

### 2.9. Gene Set Enrichment Analysis (GSEA)

GSEA analysis was performed by GSEA software (version: 4.2.3) to explore the possible biological pathways involved in CRlncSig in OC. We acquired the annotated gene set “c2.cp.kegg.v7.4.symbols.gmt” from the Molecular Signatures Database to enable the GSEA software to assess pertinent molecular mechanisms and pathways in relation to gene expression profiles and classifications. For each analysis, we conducted 1000 gene set permutations to derive a normalized enrichment score (NES), which served as the basis for ranking the pathways enriched in each phenotype. Gene sets exhibiting an adjusted *p*-value < 0.05 were deemed significant.

### 2.10. Statistical Analysis

All analyses were done using R software (version 4.1.0). Wilcoxon tests were utilized to compare the difference between the two groups, and the Kruskal–Wallis test was used to compare more than two groups. ROC analysis was performed using the “timeROC” packages (version 0.4) to assess the predictive power of prognostic model. Spearman’s correlation analysis was used to test the correlation between CRGs and lncRNAs. The KM method was used to create survival curves for progression experiments, and log-rank tests were employed to assess the significance of differences. Univariate and multivariate Cox regression analyses were implemented to identify independent predictors of OS. *p* < 0.05 was considered statistically significant.

## 3. Results

### 3.1. Identification of CRLs in OC

**Figure 1:**
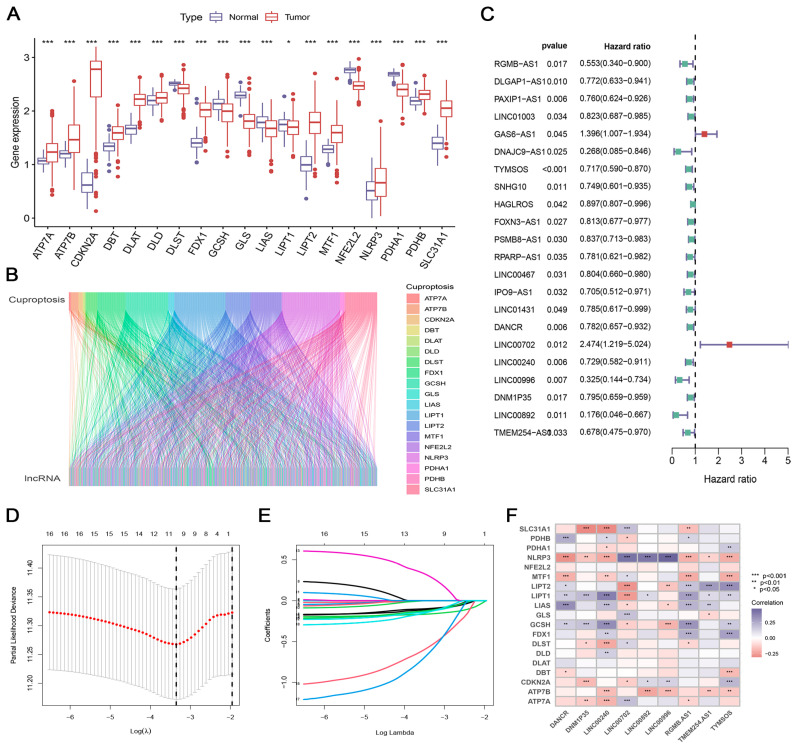
Identification of cuproptosis-related lncRNAs (CRLs) and construction of prognostic CRlncSig in OC. * *p* < 0.05; ** *p* < 0.01; *** *p* < 0.001. (**A**) The expression of 19 CRGs in OC tissues and adjacent normal tissues. (**B**) Sankey relational diagram for cuproptosis-related genes and CRLs. (**C**) HR and 95% CI of the 22 CRLs using univariate Cox regression. (**D**) A coefficient profile plot was produced against the log (lambda) sequence in the LASSO model. The optimal parameter (lambda) was selected as the first black dotted line indicated. (**E**) The trajectory of each independent variable with lambda. (**F**) Heat map of the Pearson’s correlation between the differentially expressed-lncRNAs and the differentially expressed cuproptosis-associated genes. All analyses were repeated at least three times.

A total of 379 cases of TCGA-OV, 88 cases of TCGA-GTEx normal tissue, and 273 cases of GSE9891 OC were enrolled. Nineteen CRGs were selected according to the previous reports. We detected the expression of the 19 CRGs between OC and adjacent normal tissues and observed that all CRGs were aberrantly expressed in the OC (Figure 1A). The expression of *ATP7A*, *ATP7B*, *CDKN2A*, *DBT*, *DLAT*, *DLD*, *FDX1*, *LIPT2*, *MTF1*, *NLRP3*, *PDHB*, and *SLC31A1* was significantly upregulated in OC tissues, while *DLST*, *GCSH*, *GLS, LIAS*, *LIPT1*, *NFE2L2*, and *PDHA1* were downregulated (Figure 1A), confirming the dysregulated cuproptosis process in OC. Then, a co-expression network of CRGs and lncRNAs was performed to select CRLs. In consequence, a total of 941 CRLs were identified (Figure 1B; Table S2). After intersecting the expression profiles of these CRLs with the GSE9891 cohort, 132 CRLs were identified in the training cohort.

### 3.2. Construction and Validation of the CRlncSig Based on CRLs

**Figure 2:**
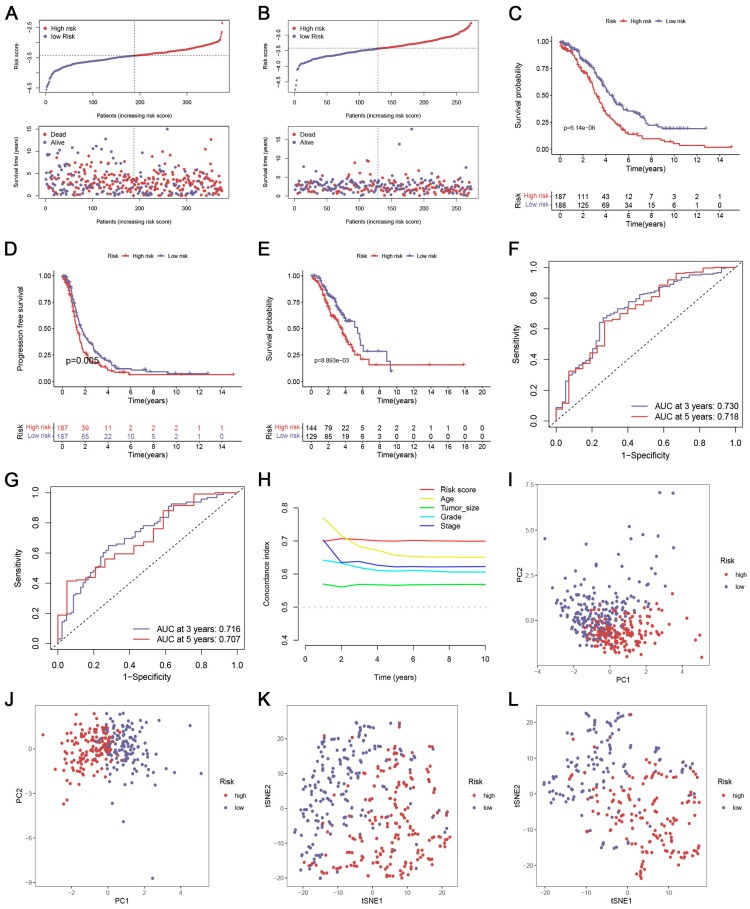
Internal and external validation of the prognostic CRlncSig. (**A**,**B**) Risk scores and survival status of each case in the training (**A**) and validation cohorts (**B**). (**C**–**E**) The OS (C) and PFS (**D**) of patients were ranked by risk score in the training cohort, and the OS (**E**) of patients was ranked by risk score validation cohort. (**F**,**G**) ROC curve for the prediction of OC survival in the training (**F**) and validation cohorts (**G**). (**H**) The concordance index (C-index) curves of the CRlncSig. (**I**–**L**) PCA and t-SNE analyses in the training (**I**,**J**) and validation cohorts (**K**,**L**). All analyses were repeated at least three times.

Based on the 132 CRLs, we performed a univariate Cox regression analysis and identified 22 OS-related CRLs (Figure 1C). Subsequently, the LASSO method further narrowed down the candidate genes, and nine CRLs with optimal λ values were screened (Figure 1D,E). In the end, the nine CRLs (RGMB-AS1, TYMSOS, DANCR, LINC00702, LINC00240, LINC00996, DNM1P35, LINC00892, and TMEM254-AS1) were selected to develop the predictive CRlncSig (Figure 1D,E). A heatmap depicting the relationships between 19 CRGs and nine CRLs was plotted in Figure 2F. Afterward, the risk score was determined as follows: The risk score = (−0.1051 × RGMB-AS1) + (−0.1623 × TYMSOS) + (−0.1284 × DANCR) + (0.2921 × LINC00702) + (−0.0956 × LINC00240) + (−0.5539 × LINC00996) + (−0.1166 × DNM1P35) + (−0.6233 × LINC00892) + (−0.1340 × TMEM254-AS1).

The distribution of risk scores was visually analyzed (Figure 2A). The Kaplan–Meier curves confirmed that patients with higher risk scores had significantly poor OS and PFS compared with patients with lower risk scores (Figure 2C,D). The tdROC analyses at 3 years (AUC = 0.730) and 5 years (AUC = 0.718) were conducted and suggested an outstanding performance of the CRlncSig in predicting OS (Figure 2F). The C-index also demonstrated the prognostic efficacy of CRlncSig was better than clinical characteristics (Figure 2H). PCA and t-SNE analyses were performed, and the result revealed a clear distribution between the two risk groups (Figure 2I,K).

### 3.3. Validation of the CRlncSig

To assess the performance of the CRlncSig in OC patients, we implemented one external GEO data set (GSE9891). As illustrated in Figure 2B, the risk of death gradually increases as the risk score increases. The K–M method demonstrated that the CRlncSig could stratify patients efficiently (Figure 2E). The ROC method also could validate the robustness of the signature in the GSE9891 cohort (Figure 2G). A clear separation of these two subgroups of patients was observed in the PCA and t-SNE analyses (Figure 2J,L).

### 3.4. Development of a Prognostic Nomogram

**Figure 3:**
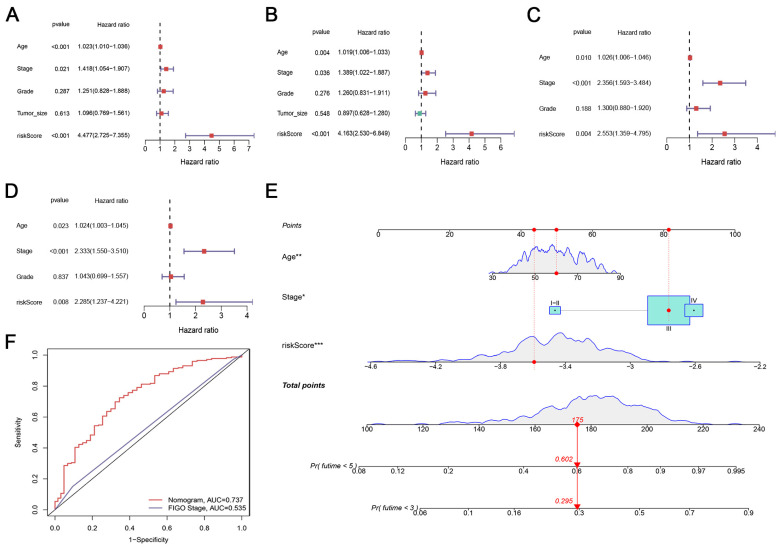

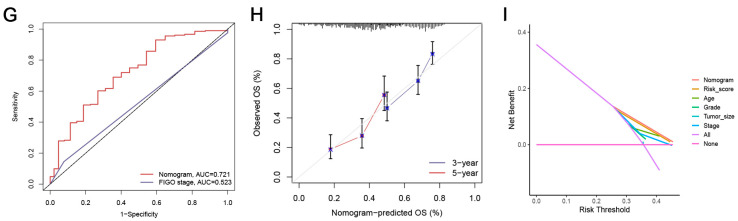
Construction and validation of nomogram. (**A**–**D**) Forest plots showing the results of the univariate and multivariate Cox analysis in the training (**A**,**B**) and validation cohorts (**C**,**D**). (**E**) Nomogram for predicting 3- and 5-year overall survival of OC patients. * *p* < 0.05; ** *p* < 0.01; *** *p* < 0.001. (**F**,**G**) Comparison of the ROC curves for predicting three- and five-year survival in the CRlncSig (**F**) and FIGO stage (**G**). (**H**) Calibration curves of the nomogram prediction of 3-year and 5-year OS of patients with OC. (**I**) Decision curve analysis (DCA) of the CRlncSig. All analyses were repeated at least three times.

Univariate and multivariate Cox analyses indicated that the CRlncSig was a powerful and independent indicator in the training (Figure 3A,B) and validation cohorts (Figure 3C,D). To facilitate the clinical practice of the CRlncSig, we developed a novel prognostic nomogram incorporating age, FIGO stage, and risk score (Figure 3E). ROC curves demonstrate that the efficacy of the nomogram was higher than that of the FIGO stage (Figure 3F,G). The calibration plot demonstrated the consistency of the predicted OS and observed OS (Figure 3H). The decision curve analysis (DCA) curve results also validated the prominent predictive efficacy of the CRlncSig (Figure 3I).

### 3.5. Immune Landscape in OC Patients with Different Risk Groups

**Figure 4:**
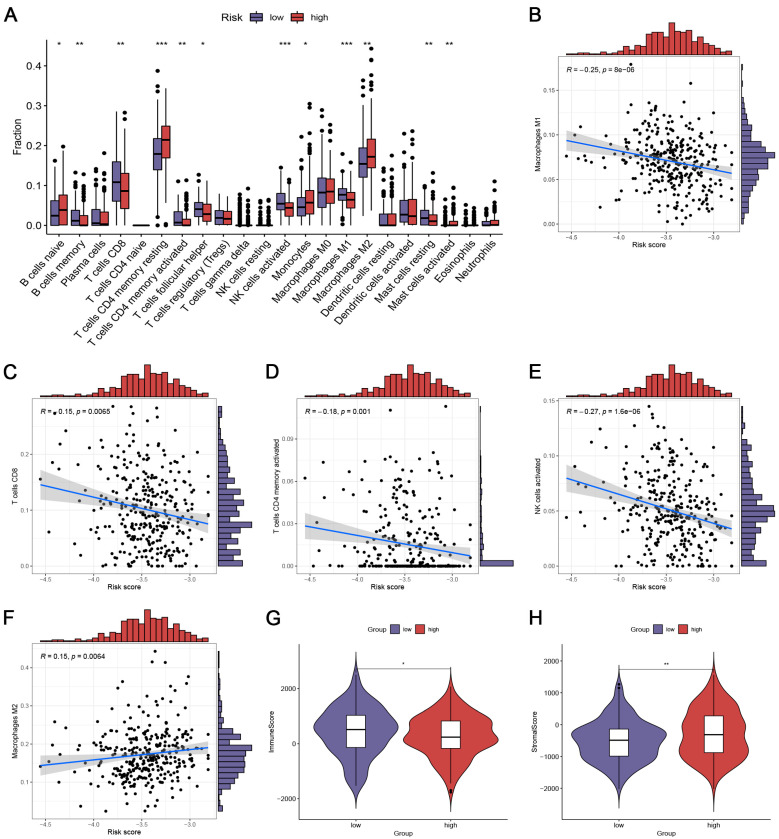
Immune infiltration status and TME in OC patients with different risk groups. (**A**) Comparison of the abundance of immunocytes between low- and high-risk groups. * *p* < 0.05; ** *p* < 0.01; *** *p* < 0.001 (**B**–**F**) Spearman’s correlation analysis between the CRlncSig and the abundance of immunocytes. (**G**,**H**) Comparison of the immune score (**G**) and stromal score (**H**) between low- and high-risk groups. R: Spearman coefficient. All analyses were repeated at least three times.

To investigate the correlation between the CRlncSig and antitumor immunity in OC, the CIBERSORT algorithm was employed to evaluate the component disparity of immunocytes in OC patients. We discovered remarkably higher levels of CD8+ T cells, activated memory CD4+ T cells, M1 macrophages, activated NK cells, memory B cells, and mast cells resting (Figure 4A; *p* < 0.05). In contrast, the high-risk subgroup exhibited increased expression of M2 macrophages, activated mast cells, resting memory CD4+ T cells, naïve B cells, and monocytes (Figure 4A; *p* < 0.05). Additionally, we found remarkable correlativity between the risk score and multiple immunocytes such as M1 macrophages (R = −0.25, *p* < 0.001), CD8+ T cells (R = −0.15, *p* = 0.006), activated memory CD4+ T cells (R = −0.18, *p* = 0.001), activated NK cells (R = −0.27, *p* < 0.001), and M2 macrophages (R = 0.15, *p* = 0.006) (Figure 4B–F). Through the ESTIMATE algorithm, we quantified the entire infiltrations of immune and stromal cells between the two risk groups. Our results showed that the low-risk group had a higher immune score, while the high-risk group had a higher stromal score (Figure 4G,H).

### 3.6. Immunotherapeutic Response Prediction

**Figure 5:**
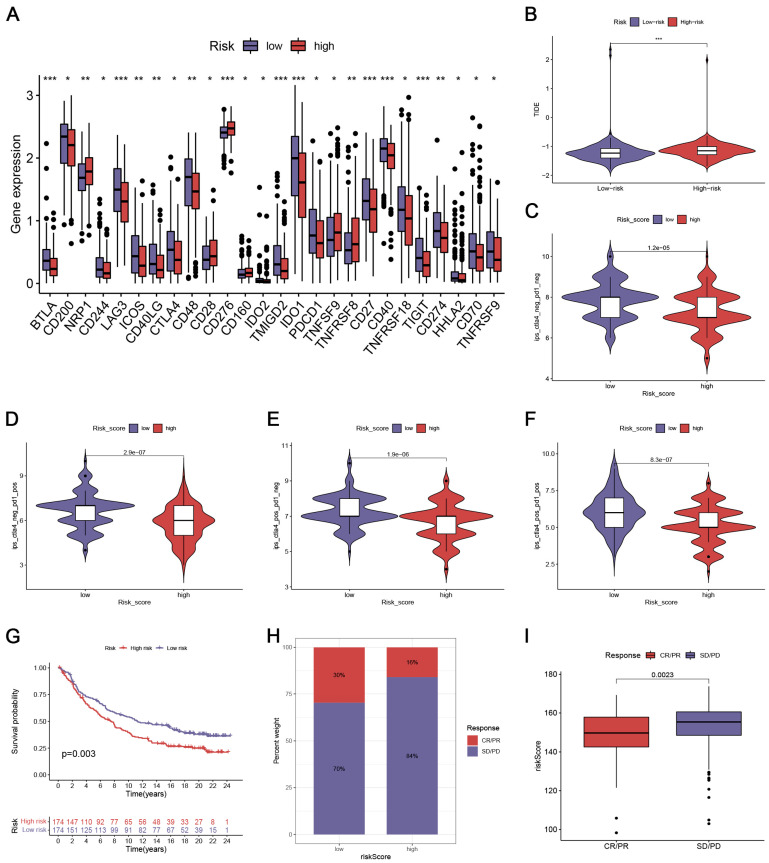
Comparison of immunotherapeutic response between high- and low-risk groups. (**A**) Comparison of the expression of immune checkpoints in the high- and low-risk groups. * *p* < 0.05; ** *p* < 0.01; *** *p* < 0.001 (**B**) Comparison of the tumor immune dysfunction and exclusion (TIDE) score between different risk groups. (**C**–**F**) Relative distribution of immunophenoscore (IPS) in high- and low-risk groups. (**G**) Kaplan–Meier survival curves of patients with different risk scores in the anti-PD-L1 immunotherapy cohort. (**H**) The proportion of patients with response to immunotherapy in different risk groups. CR, complete response; PR, partial response; SD, stable disease; PD, progressive disease. (**I**) Distribution of risk scores in different anti-PD-L1 clinical response groups. All analyses were repeated at least three times.

We further looked at the value of the CRlncSig in predicting immunotherapeutic response. We first compared the expression of major immune checkpoints in two risk groups. The result indicated that the expression of HAVCR2, PD-1, PD-L1, and CTLA4 was higher in the low-risk group than those in the high-risk group (Figure 5A). We further used TIDE to assess the response to immunotherapy in the two risk groups. Patients in high-risk groups led a statistically significant increase in TIDE score (Figure 5B), which means their tumors were more likely to acquire immune escape. To explore the correlation between CRlncSig and immunotherapy response, we examined the IPS scores after anti-CTLA-4/PD-1 therapy, and we found that the low-risk score group had higher IPS scores (Figure 5C–F), indicating that the low-risk group could achieve better immunotherapy results. We next evaluated the prognostic value of the risk score for immune checkpoint therapy by assigning patients to the IMvigor210 cohort. The prognosis of patients with low-risk scores was significantly better than those with high-risk scores (Figure 5G). Patients in the low-risk subgroup had a higher response rate than those in the high-risk subgroup (Figure 5H), and patients who responded to immunotherapy had lower risk scores (Figure 5I).

### 3.7. Correlation Analysis of the CRlncSig with TMB

**Figure 6:**
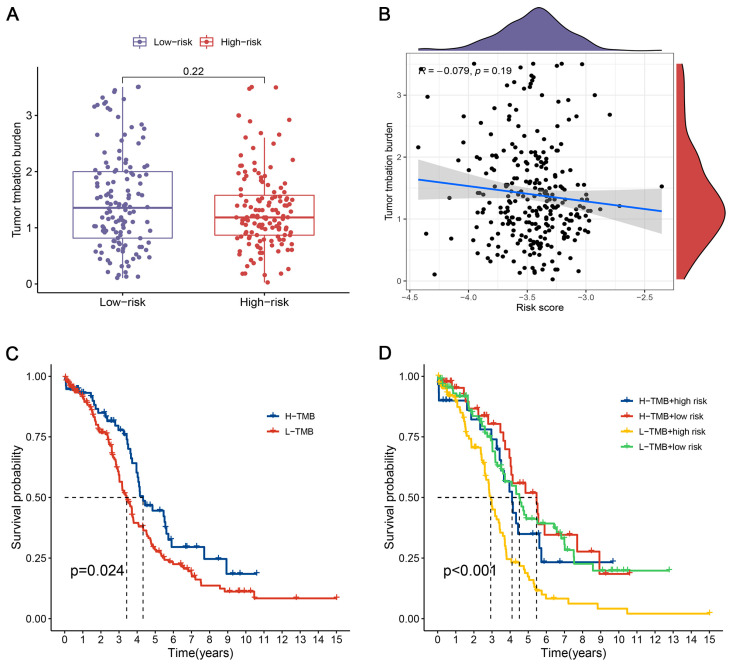
Correlation analysis of the CRlncSig with tumor mutational burden (TMB). (**A**) The differences in TMB in patients with CRC between the high- and low-risk groups. (**B**) Correlation between TMB and risk score. (**C**) Survival analysis in high and low TMB groups. (**D**) The K–M survival analysis of OC patients regarding TMB combined with CRlncSig. R: Spearman coefficient. All analyses were repeated at least three times.

We derived the TMB scores of OC patients based on the somatic mutation data of TCGA. We noted that the high low-risk score group had a greater TMB than the low low-risk score group and that the risk score was negatively correlated with TMB, but not significantly different (Figure 6A,B). Moreover, patients in the low TMB group had poorer OS (Figure 6C). Likewise, we discovered that the low-risk score group with a high TMB demonstrated greater survival (Figure 6D).

### 3.8. Drug Sensitivity Prediction

**Figure 7:**
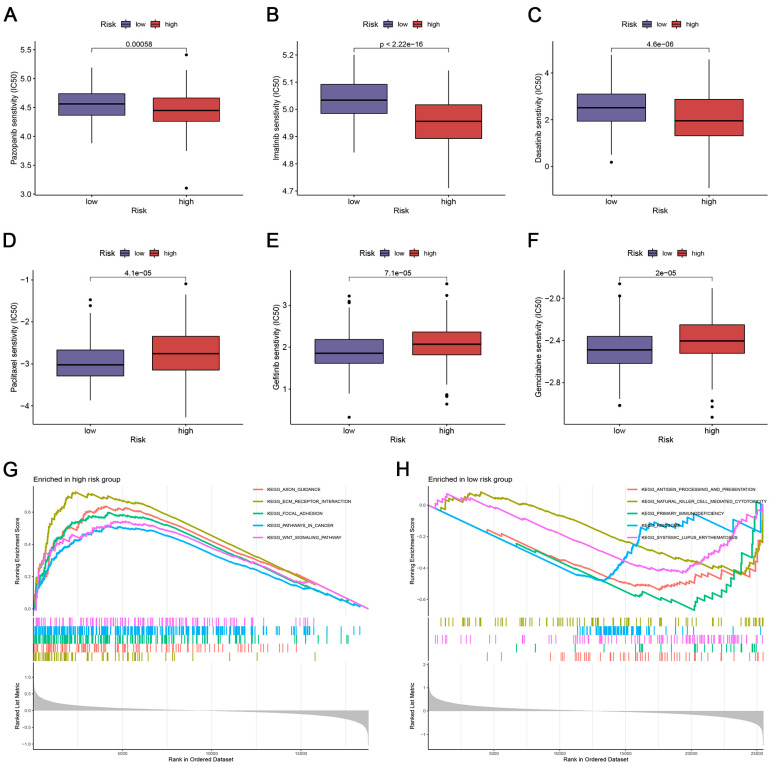
Drug sensitivity and pathway enrichment analyses. (**A**–**F**) Comparison of susceptibility to IC50 in patients with a risk score. (**G**,**H**) Significantly enriched KEGG pathways in OC patients with high- (**G**) and low-risk groups (**H**) were analyzed using GSEA software. All analyses were repeated at least three times.

To further investigate whether the risk score could predict drug sensitivity in OC patients, we compared IC50 levels in two groups of some chemotherapeutic drugs or inhibitors commonly used in a clinical setting. We found that the high-risk score group had lower IC50s for Pazopanib, Imatinib, and Dasatinib, indicating that patients with high-risk scores benefited more from these three drugs (Figure 7A–C). Meanwhile, Paclitaxel, Gefitinib, and Gemcitabine may have a better potential for treating low-risk patients (Figure 7D–F).

### 3.9. Functional Analysis of the CRlncSig

The GSEA of the high-risk group was mainly enriched in the stromal and cancer-related pathways, including ECM receptor interaction, focal adhesion, pathways in cancer, and the WNT signaling pathway (Figure 7G). Meanwhile, the GSEA of the low-risk group was mainly enriched in the immune response, including natural killer cell-mediated cytotoxicity, antigen processing and presentation, and primary immunodeficiency (Figure 7H).

## 4. Discussion

OC is a heterogeneous group of neoplasm with distinct clinicopathological and molecular features and prognosis [25]. Despite optimal surgery and appropriate first-line chemotherapy, many patients with OC will experience a recurrence and chemotherapy resistance [26], and the five-year survival rate is approximately 45% [27]. Therefore, a possible breakthrough in improving the outcomes and extending the survival of patients with OC could result from the development of new and practicable prognostic biomarkers and treatment targets. In recent years, researchers have discovered dozens of RCD modes, among which apoptosis, ferroptosis, pyroptosis, and necroptosis are the most widely studied in OC [28,29,30]. Cuproptosis is a copper-triggered modality of mitochondrial cell death distinct from other types of cell death [9]. As a new type of RCD, cuproptosis can serve as an alternative and attractive therapeutic for supporting anticancer immunity [9]. Recent studies have begun to elucidate the intricate connections between cuproptosis, mitochondrial function, and lncRNAs, emphasizing the need for a comprehensive understanding of these relationships. For instance, research has highlighted the role of CRLs in cancer, revealing that alterations in mitochondrial respiration can influence tumor behavior and patient prognosis. In uterine corpus endometrial carcinoma, a model incorporating CRLs was developed to enhance prognostic predictions and guide treatment strategies [31]. These findings are echoed in hepatocellular carcinoma, where a novel lncRNA signature related to cuproptosis was shown to predict patient prognosis and immunotherapy effectiveness, highlighting the interplay between cuproptosis and immune responses in malignancies [32]. Furthermore, the implications of cuproptosis extend beyond specific cancer types. A comprehensive analysis in pancreatic adenocarcinoma revealed that cuproptosis-related lncRNAs could stratify patients based on their prognostic outcomes, emphasizing the importance of mitochondrial respiration in tumor dynamics [33]. The study also demonstrated that certain lncRNAs could modulate glycolytic pathways, thus influencing cancer cell proliferation and survival, which is a critical aspect of tumor metabolism. Overall, the integration of cuproptosis with mitochondrial respiration and lncRNA dynamics presents a promising avenue for advancing cancer therapies. Future research should focus on elucidating the specific metabolic pathways involved in cuproptosis and the regulatory roles of lncRNAs, as these insights could lead to novel therapeutic strategies aimed at targeting metabolic vulnerabilities in cancer cells.

The application of bioinformatics in tumor research is becoming increasingly widespread, mainly reflected in data analysis and the discovery of biomarkers. Through high-throughput sequencing technology, researchers can deeply analyze the genomic characteristics of tumors, thereby revealing the mechanisms of tumor occurrence and development. Recent advancements in computational techniques, particularly atomistic molecular dynamics and density functional tight binding (DFTB), have significantly enhanced our understanding of molecular interactions in biological systems, including cancer [34]. These methods allow researchers to simulate the dynamic behavior of biomolecules at an atomic level, providing insights that can inform experimental design and therapeutic strategies. Moreover, the integration of these advanced simulation techniques with experimental data can lead to a more nuanced understanding of the molecular mechanisms underlying cancer progression and treatment resistance. By elucidating the structural and energetic aspects of molecular interactions, researchers can identify novel therapeutic targets and design more effective inhibitors. This study mainly focused on developing a prognostic CRlncSig using a biological approach, and clarified the important role of the CRlncSig in clinical outcomes, immune landscape, response prediction to different therapies, and tumor mutation landscape, which allowed for accurately evaluating the prognosis of OC patients and developing more effective therapeutic strategies. First, we comprehensively analyzed the expression profile of 19 CRGs via the integrated analysis of TCGA and GTEx databases. These CRGs are all expressed aberrantly in OC, indicating cuproptosis is involved in the progression of OC. Then, correlation analysis identified 132 CRLs and univariate Cox analysis revealed 22 prognostic CRLs. Through LASSO regression analysis, a novel prognostic CRlncSig integrating nine CRLs was generated. In the training cohort, we observed an obvious difference in OS between the two groups. This result was confirmed in an independent GEO cohort, indicating a good reproducibility of this CRlncSig in OC. Compared with traditional FIGO staging, it showed better prognostic predictive power in OC patients. The signature was confirmed as an independent prognostic factor in OC patients. Additionally, we also constructed a CRlncSig-based nomogram, which can predict the prognosis of OC patients more accurately and increase the clinical utility. Moreover, we observed obvious differences in immune cell subpopulations, TME characteristics, expression of the immune checkpoint, and immunotherapeutic response between the two risk groups. The predictive value of the risk score for checkpoint immunotherapy was also confirmed in the IMvigor210 cohort.

In the present study, the CRlncSig was constructed based on nine CRLs, including RGMB-AS1, TYMSOS, DANCR, LINC00702, LINC00240, LINC00996, DNM1P35, LINC00892, and TMEM254-AS1. Some of these lncRNAs have been reported in previous literature. For instance, lncRNA DANCR (also known as ANCR) is a cancer-associated lncRNA, and dysregulation of DANCR affects cancer cell proliferation, apoptosis, migration, and invasion through different mechanisms, including acting as a miRNA sponge, stabilizing mRNA, and interacting with proteins [35]. Pei et al. [36] observed that DANCR expression was elevated in OC tissues and accelerated the proliferation and migration of OC cells by negatively regulating UPF1 expression. Similarly, DANCR as an oncogene promotes OC progression via targeting miR-145 [37]. LINC00702 can promote the progression of OC by interacting with EZH2 to suppress the transcription of KLF2 [38]. LncRNA RGMB-AS1 has been reported to play oncogenic or anti-oncogenic roles in the tumorigenesis and progression of various malignancies [39,40,41]. Zhang et al. [42] demonstrated that lncRNA RGMB-AS1 can inhibit malignant behavior and EMT in nasopharyngeal carcinoma by binding to forkhead box A1. Evidence confirmed that LINC00240 can enhance cervical cancer progression by inducing miR-124-3p/STAT3/MICA-mediated natural killer T (NKT) cell tolerance [43]. As the roles of most lncRNAs in the CRlncSig have not yet been reported in OC, our findings may provide some useful insights for further in-depth studies. Further research is required to disclose the roles of the above nine lncRNAs in the development of OC via a cuproptosis mechanism.

GSEA could assist in the investigation of the underlying functions through the subtle expression changes of multiple genes. As illustrated in Figure 7G,H of GSEA, the low-risk group seemingly harbored immune activation pathways, while the high-risk group mainly enriched in stromal and carcinogenesis pathways. Accumulating evidence has revealed crosstalk between copper metabolism and antitumor immunity [44]. Florida et al. [44] verified that copper supplementation promoted the expression of PD-L1 mRNA and protein levels in cancer cells. RNA sequencing showed that copper regulates key signaling pathways mediating PD-L1-driven cancer immune escape.

However, no clear studies have shown a direct relationship between cuproptosis and TME in OC. After enriching several immune-related pathways, we compared the different activations of anticancer immune responses between two risk groups with OC. The TME score between the two groups was significantly different. The low-risk score was correlated with immune activation and the high-risk score exhibited a significant stroma activation status. Through TME immune infiltration analysis, we found that the risk score exhibited remarkably negative correlations with CD8+ T cells, M1 macrophages, activated memory CD4+ T cells, and activated NK cells. Many studies have confirmed that the infiltration of the CD4+ T cell and CD8+ T cell are significantly correlated with a better prognosis for patients with OC [45]. The effect of adoptive T cell therapy in the treatment of OC has been confirmed by phase I experiments, although most patients can only maintain a short response due to T cell inhibition or depletion [46]. Moreover, a positive correlation between risk scores and M2 macrophages, activated mast cells, and monocytes was observed. The infiltration of high-density M2-like tumor-associated macrophages (TAMs) in OC is predictive of poor prognosis [47]. It can secrete various cytokines, chemokines, enzymes, and exosomes to microRNAs, thereby inducing the progression and chemoresistance of OC [47]. M2-like TAMs can promote early transcoelomic metastasis of OC in the peritoneum by assisting the spheroid formation and cancer cell attachment to the metastatic area—the omentum [48]. Overall, the weakened anti-tumor immunity in the high-risk group may explain the poor prognosis of OC patients.

Immunotherapy has become a new promising approach for treating OC, especially immune checkpoint inhibitors (ICIs), which have become an effective treatment [49,50]. Checkpoint inhibitor therapy, such as PD-1 and PD-L1, has achieved good clinical efficacy in OC [51,52]. In addition, several trials of the PD-1/PD-L1 blockade in OC are underway, including in combination with antiangiogenic or targeted PARP inhibitors [53,54,55]. In patients with OC, the clinical effect of the combined application is usually superior to that of a single immune checkpoint blockade [56]. We found that the expression of HAVCR2, CTLA4, PD-1, and PD-L1 was remarkably elevated in the low-risk group, indicating that low-risk patients may respond more readily to immunotherapy. We further found that the high-risk group has a higher TIDE score, suggesting a greater likelihood of immune escape. The IPS score further demonstrated a poor immunotherapy response in the high-risk group, which is consistent with previous findings. Evidence suggests that patients with a high TMB status demonstrate durable clinical responses to immunotherapy. In this work, the low-risk score group was significantly correlated with higher TMB and was more sensitive to anti-CTLA4 and anti-PD-1 therapy. Finally, an immunotherapeutic cohort (IMvigor210) confirmed the value of the CRlncSig in predicting immunotherapy outcomes. Taken together, patients with low-risk scores may benefit more from immunotherapy.

This investigation is not without its drawbacks. First, the data for constructing the CRlncSig are all from public databases. Therefore, the expression of these CRlncSig CRLs needed to be validated in clinical specimens. Second, the number of samples we retrieved from the TCGA database and GEO database is relatively small, and the reliability of the CRlncSig established in this study needs to be further validated by including a larger sample size of clinical trials. Third, since cuproptosis was newly discovered, some CRLs we identified have not been reported yet, especially in immunotherapy, thus basic experiments to investigate their functions are needed.

## 5. Conclusions

We constructed an effective CRlncSig and nomogram, which may contribute to risk stratification and prognostic assessment. The CRlncSig performed excellently in assessing TME characteristics and immunotherapy outcomes in OC patients, suggesting its potential application in future immunotherapy.

## Data Availability

All data that support this manuscript are available in the public databases TCGA database (http://www.cancer.gov/tcga, accessed on 10 August 2022) and Gene Expression Omnibus (https://www.ncbi.nlm.nih.gov/gds/, accessed on 10 August 2022).

## Supplementary Materials

The following supporting information can be downloaded at: https://www.mdpi.com/article/10.3390/biomedicines12112640/s1, Table S1: Summary of 19 cuproptosis-related genes; Table S2: Spearman correlation analysis between the cuproptosis-related genes and lncRNAs.
